# The value of 7 peripheral blood serum ratios in diagnosis and prediction of disease activity of patients within inflammatory bowel disease individuals

**DOI:** 10.3389/fmed.2023.1122005

**Published:** 2023-04-05

**Authors:** Jun Pan, Jiao Li, Yuanjun Gao

**Affiliations:** ^1^Department of Gastroenterology, Taihe Hospital, Hubei University of Medicine, Shiyan, Hubei, China; ^2^Department of Gastroenterology, Renmin Hospital of Wuhan University, Wuhan, Hubei, China

**Keywords:** chronic inflammatory ratios, neutrophil to pre-albumin ratio (NPAR), inflammatory bowel disease, IBD-related neoplasia (IBDN), serum biomarker

## Abstract

**Objective:**

In recent years, a number of studies have suggested that inflammation-based biomarkers can be applied in the diagnostics and prognostic testing of disease. However, the association between these ratios and inflammatory bowel disease (IBD) remains unclear. We aimed to investigate the role of these inflammation-based ratios in patients with IBD.

**Methods:**

Retrospective analysis of 362 patients with IBD and 100 healthy individuals from January 2016 and December 2021. The receiver operating characteristic curve and logistic regression analysis was applied to explore the diagnostic and predictive performance of the seven ratio markers [neutrophil- to-albumin ratio (NAR), neutrophil-to-pre-albumin ratio (NPAR), albumin-to-alkaline-phosphatase ratio (AAPR), albumin-to-globulin ratio (AGR), albumin-to-fibrinogen ratio (AFR), fibrinogen-to-pre-albumin ratio (FPR), and Prognostic Nutritional Index (PNI)] regarding to disease activity in IBD individuals.

**Results:**

Compared with healthy controls, patients with Crohn’s disease (CD) or ulcerative colitis (UC) exhibited higher levels of NAR, NPAR, FPR (*P* < 0.001), lower levels of AAPR, and PNI (*P* < 0.001). Multivariate logistic regression showed that the level of NPAR (OR = 1.12, 95%CI: 1.02–1.23, *P* = 0.016) and AGR (OR = 1.01, 95%CI: 1.01–1.12, *P* < 0.001) was an independent risk factor of IBD. Then, we found the level of NPAR (OR = 1.10, 95%CI: 1.01–1.20, *P* = 0.02) and PNI (OR = 0.83, 95%CI: 0.71–0.96, *P* = 0.01) was independently associated with disease activity. Besides, a positive association was observed between the level of NPAR and two clinical scores [Harvey Bradshaw index (HBI) in patients with CD, Mayo score in patients with UC]. Finally, the level of NPAR (*P* = 0.002) and PNI (*P* = 0.003) showed a significant difference in the IBD-associated neoplasia group and IBD without neoplasia group.

**Conclusion:**

Our data first suggests NPAR as a putative biomarker for diagnosing and predicting disease activity in patients with IBD. Investigations involving a larger number of IBD individuals are necessary to validate its use as an easily obtained peripheral blood biomarker of IBD.

## Introduction

Inflammatory bowel disease (IBD) is a chronic, non-specific, inflammatory bowel disorder, including Crohn’s disease (CD), ulcerative colitis (UC), unclassified colitis, and indeterminate colitis (1). However, CD and UC are the two principal subtypes of IBD and their pathogenesis is remained unclear. The characteristics of easily relapse and large out-of-pocket healthcare expenses severely affect both the mental and physical quality of life of patients with IBD (2). Timely diagnosis and identification of disease activity can aid physicians in selecting suitable treatment strategies (3). The current method for diagnosis and predictive disease activity of IBD are mainly endoscopic and biopsy, with the drawbacks of invasive and high economic consumption. Thus, more studies are needed to find an efficient, cost-effective, and easily obtained biomarker for clinical practice.

Recently, studies have suggested inflammation and nutrition conditions are closely associated with the initiation and progression phase of IBD (4). It is reasonable to infer that peripheral blood chronic inflammatory ratios may be of use in IBD clinical practice. To date there have been several studies indicating C-reactive protein-to-albumin Ratio (CRP/ALB, CAR) as a chronic inflammatory ratio has potential use in both IBD diagnostics (5) and many types of cancer prognostication (6). Some studies have shown that the neutrophil-to-albumin/pre-albumin ratio (NAR/NPAR) (7, 8), albumin-to-alkaline -phosphatase ratio (AAPR) (9), albumin-to-globulin ratio (AGR) (10), albumin-to-fibrinogen ratio (AFR) (11), fibrinogen-to-pre-albumin ratio (FPR) (12), and prognostic nutritional index (PNI) (13) can assist diagnosis or predict the clinical outcomes of malignant tumor. However, no study has reported the association between these peripheral blood ratios and IBD.

Many studies have indicated that disease duration is linked to an increased risk of IBD-related colorectal cancer (CRC), however, the pathological mechanisms remain ambiguous (14). Daniel et al. (15, 16) reported that EYA4, SLIT2, abnormal DNA ploidy, and p53 immunopositivity are important risk factors for developing colorectal neoplasia in high-risk IBD patients. So far, studies of unearthing easy-to-use prognostic biomarkers which can be applied to evaluate the risk of dysplasia of IBD are lacking.

In this study, for the first time, we evaluated the potential clinical significance of seven peripheral blood ratios regarding to diagnosis and prediction of disease activity within IBD patients in real-world practice. Furthermore, we explored the potential use of the seven ratios in predicting IBD-related neoplasia (IBDN), to facilitate individualized prognosis prediction and clinical decision-making.

## Patients and methods

### Study population

All the confirmed adult IBD patients (outpatient and inpatient Department of Internal Medicine) at Renmin Hospital of Wuhan University were consecutively recruited between January 2016 and December 2021. In this retrospective, a total of 362 patients confirmed with IBD (202 with CD, 160 with UC) have been included. The participants who visited the Health Examination Center at the same given period were assigned to the control group, which consisted of 100 health controls (HC) after matched age and sex. This study was conducted in accordance with the ethical principles of the Declaration of Helsinki and in compliance with Renmin Hospital of Wuhan University (No. 2018K-C089). Additionally, all patients will be informed of the research objectives and have signed the consent before participating in the study.

### Inclusion and exclusion criteria

The IBD diagnosis was made by an experienced physician and was based on endoscopic, radiological, histological, and clinical criteria. In line with the Montreal criteria, we made a diagnosis and classification of clinical phenotypes (17).

Inclusion criteria: (a) Patients with a diagnosis of IBD, 13-80 years, no patients from the Pediatric department; (b) Patients who have undergone continuous digestive tract endoscope in our institute; and (c) patients agreed to participate in the study.

Exclusion criteria: (a) patients under any experimental therapies (i.e., in clinical trials); (b) patients with missing information; (c) Patients with intestinal tuberculosis, ischemic bowel disease, intestinal ulcers caused by non-steroidal anti-inflammatory drugs, and radiation enteritis; (d) patients with signs of sepsis, hemolytic anemia, hemochromatosis, and chronic kidney disease; (e) Patients with other autoimmune diseases; and (f) Patients are pregnant women.

### Clinical data collection

At our institution, patients’ entire data were entered into a clinical computerized medical record system. It will be updated after patients each visit no matter in outpatient or inpatient clinic. We collected the baseline information of each enrolled subject, including age, sex, duration of disease, symptoms, medications, and colonoscopy with histopathological results. A total of 50 variables derived from peripheral blood biochemical data, such as pre-albumin and alkaline phosphatase (ALP), were retrieved from the electronic medical record system. A total of 28 variables derived from the complete blood count, such as white blood cell count (WBC) and red cell distribution width (RDW) were collected. In addition to this, we included some typical meaningful indicators, such as erythrocyte sedimentation rate (ESR) and fecal calprotectin (Fc) during the study period. All the chronic inflammatory ratios were calculated according to standard formulas. For the benefit of the analysis, several ratios amount were recorded in *100 or *1000.

### Assessment of clinical disease activity of IBD

Scores for clinical activity of disease were calculated by reviewing the clinical records of each included patient. Each case activity was evaluated by two experienced doctors according to a widely accepted rating scale [CD: Crohn’s disease activity index (CDAI) (18) and Harvey Bradshaw index (HBI) (19); UC: partial Mayo score (20)].

As for patients with CD, the active disease was defined as CRP ≥ 5mg/L and/or CDAI > 150 points and/or HBI > 4 points. For patients with UC, having a CRP ≥ 5 mg/L and/or Mayo UC score ≥ 5 points were considered to as active phase, whereas patients having a Mayo score ≤ 4 were defined as the remission group.

### Statistical analysis

Data were analyzed using Microsoft Excel 2019 (Microsoft, version 16.66.1), SPSS software 25.0 (IBM Corp, Armonk, NY, USA), and GraphPad Prism 8.2.1 (GraphPad Software Inc., San Diego, CA, USA). Continuous variables are expressed as the mean (SD) or median (IQR), and frequency (%) for categorical variables. Differences between the levels of each variable in UC, CD, all IBD, and HC were analyzed by the Mann–Whitney U test. The difference between remission and active disease groups was also analyzed using Mann–Whitney test. Logistic regression was performed to evaluate the association between peripheral blood parameter levels and the risk of IBD. All univariate *P* values less than 0.05 and the area under the receiver operating characteristic curve (AUC) ≥ 0.6 variables were taken into further multivariable logistic regression analyses. ROC analyses were used to define the optimal cut-off value (Youden index = highest sum of sensitivity + specificity-1) for each indicator. Similar analyses were conducted to examine the correlation between peripheral blood parameters level and stage of IBD (inactive or active). In addition, a Pearson’s correlation analysis was performed to assess the relationship between significant parameters and HBI, and Mayo UC scores, respectively. The chi-square test and Fisher’s exact tests were used to analyze the significant difference in the comparisons. All analyses were 2-sided, and statistical significance was set at *P* < 0.05.

## Results

### Clinical characteristics of included participants

**FIGURE 1:**
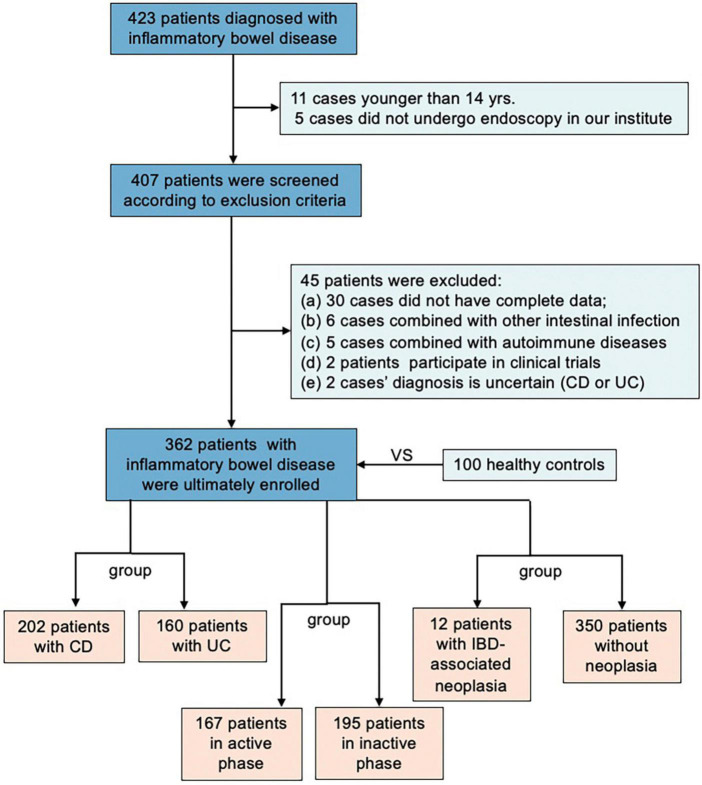
The flowchart of screening eligible patients and grouping subjects in the present study.

As shown in Figure 1, 362 patients with IBD (100 from health control, 202 from CD, and 160 from UC) were included in our study according to the inclusion and exclusion criteria. The baseline characteristics of the included IBD patients were described in Table 1. 242 (67%) were male, and the mean age of the whole cohort was 40 years (range 13–77 years). In the CD group and UC group, the mean age of the group was 36 (14–70 years), 46 (13-77 years), and 135 (67%), 107 (67%) were male, respectively.

**TABLE 1:** Study demographics, Montreal classification, and disease behavior for inflammatory bowel diseases (IBD) patient samples in this study.

Characteristics	Total IBD	CD	UC
Total no. of patients	362	202(55.8%)	160(44.2%)
Age (years)	40 ± 15.0(13–77)	36 ± 13.2(14–70)	46 ± 15.2(13–77)
Male, *n*(%)	242(67%)	135(67%)	107(67%)
Current smokers, *n* (%)	45(12.4%)	23(11.4%)	22(13.8%)
infection *Clostridium difficile*	69(19.1%)	38(18.8%)	31(19.4%)
Duration of disease (median, months)	36(0–360)	33(0–340)	41(0–360)
Abscess or fistula	35(9.7%)	30(14.9%)	5(3.1%)
HBsAg (positive)	24(6.6%)	15(7.4%)	9(5.6%)
Montreal A (1:2:3)		13(6.4%):130(64.4%): 59(29.2%)	
Montreal L (1:2:3)		72(35.6%):40(19.8%): 90(44.6%)	
Montreal B (1:2:3)		122(60.4%):52(25.7%): 28(13.9%)	
Montreal E (1:2:3)			23(14.4%):43(26.9%): 94(58.8%)
Previous intestinal surgery	15(4.1%)	12(5.9%)	3(1.9%)
**Disease activity**			
Active	167(46.1%)	91(45.0%)	76(47.5%)
Inactive	195(53.9%)	111(55.0%)	84(52.5%)
**Medications**			
Steroid therapy	78(21.5%)	48(23.8%)	30(18.8%)
Aminosalicylate therapy	294(81.2%)	171(84.7%)	123(76.9%)
Immunosuppressive therapy	108(53.5%)	87(43.1%)	21(13.1%)
Biologics	125(34.5%)	101(50.0%)	24(15.0%)

UC, ulcerative colitis; CD, Crohn’s disease. Montreal A: 1 = age ≤ 16 years at diagnosis, 2 = 17–40 years, 3 = age > 40 years. Montreal L:1 = location ileum, 2 = colonic, 3 = ileocolonic. Montreal B: 1 = non-stricturing, non-penetrating, 2 = stricturing, 3 = penetrating; Montreal E: 1 = ulcerative proctitis, 2 = left sided (distal) ulcerative colitis, 3 = pancolitis. Montreal E:1 = proctitis, 2 = left-sided colitis, 3 = extensive colitis.

### Seven peripheral blood ratios in healthy controls and IBD patients

Levels of seven ratios were analyzed (Table 2). Among these ratios, NAR, NPAR, and FRP were found to have significantly higher levels in all IBD cohorts (both in CD and UC groups) compared to the healthy controls. On the contrary, the levels of AAPR and PNI were lower in all IBD cohorts (both in CD and UC groups) compared to the healthy controls. AGR has a significantly higher level in CD groups, but lower in UC groups. No significant difference in AFR levels were observed between UC/CD group and healthy controls group.

**TABLE 2:** The seven ratios in healthy controls, CD, UC, and all IBD patients.

7 Ratios	Healthy controls (*N* = 100)	CD (*N* = 202)	UC (*N* = 160)	All IBD (*N* = 362)
**NAR: Neutrophil/albumin ratio × 100**				
Mean(SD)	8.14(3.17)	15.54(31.41)	11.74(6.65)	13.97(24.50)
Median(range)	7.91(4.25–17.78)	10.04(2.36–395.92)	10.51(3.01–32.24)	10.17(2.36–395.92)
*P*-value (CD/UC/All IBD: controls)	–	**<0.001**	**<0.001**	**<0.001**
**NPAR: Neutrophil/pre-albumin ratio × 1000**				
Mean(SD)	14.15(6.74)	31.96(28.60)	30.42(26.02)	31.33(27.54)
Median(range)	13.58(6.46–49.90)	21.53(0.45–188.43)	21.77(5.19–123.27)	21.74(0.45–188.43)
*P*-value (CD/UC/All IBD: controls)	–	**<0.001**	**<0.001**	**<0.001**
**AAPR: Albumin/alkaline phosphatase ratio**				
Mean(SD)	0.63(0.16)	0.54(0.20)	0.53(2.60)	0.54(0.18)
Median(range)	0.60(0.33–1.08)	0.54(0.02–1.35)	0.51(0.15–0.99)	0.52(0.02–1.35)
*P*-value (CD/UC/All IBD: controls)	–	**<0.001**	**<0.001**	**<0.001**
**AGR: albumin/globulin ratio**				
Mean(SD)	1.91(0.23)	6.14(6.18)	1.68(0.40)	4.20(5.15)
Median(range)	1.89(1.33–2.69)	5.06(0.06–53.87)	1.66(0.89–3.46)	3.67(0.06–53.87)
*P*-value (CD/UC/All IBD: controls)	–	**<0.001**	**<0.001**	**<0.001**
**AFR: Albumin/fibrinogen ratio**				
Mean(SD)	0.19(0.04)	0.20(0.17)	0.19(0.03)	0.20(0.13)
Median(range)	0.19(0.08–0.27)	0.19(0.09–2.42)	1.66(0.89–3.46)	0.19(0.09–3.46)
*P*-value (CD/UC/All IBD: controls)	–	0.42	0.29	0.44
**FPR: Fibrinogen/pre-albumin ratio × 100**				
Mean(SD)	98.56(32.96)	223.63(930.04)	133.68(72.33)	186.64(715.71)
Median(range)	90.90(52.20–274.70)	114.45(6.91–10602.56)	109.04(54.02–581.42)	114.30(6.91–10602.56)
P value (CD/UC/All IBD: controls)	–	**<0.001**	**<0.001**	**<0.001**
**PNI:(Albumin (g/L) + (5 × total lymphocyte count)**				
Mean(SD)	50.97(6.20)	45.18(11.07)	46.54(9.49)	45.74(10.45)
Median(range)	51.35(6.50-66.50)	47.08(4.40-65.10)	47.23(12.94-69.98)	47.10(4.40-69.98)
P value (CD/UC/All IBD: controls)	–	**<0.001**	**<0.001**	**<0.001**

Bold fonts represented that P value was < 0.05.

**FIGURE 2:**
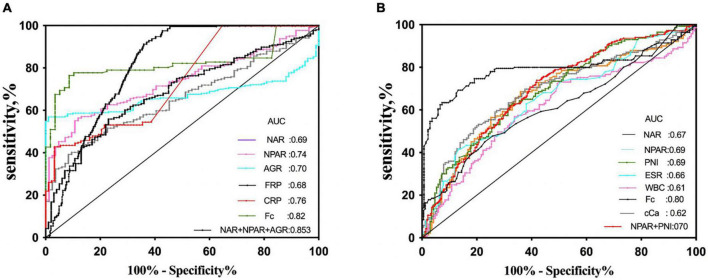
Receiver operating curve analysis (ROC) of inflammatory- based markers in differentiating inflammatory bowel diseases (IBD) from the control group **(A)**; active clinic stage from inactive group in patients with IBD **(B)**.

**FIGURE 3:**
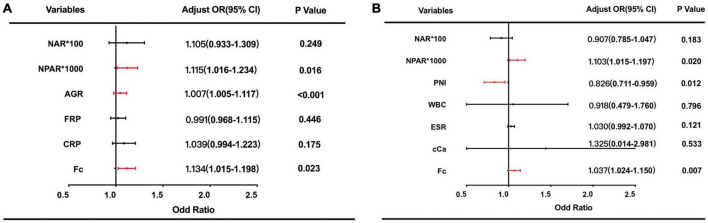
ORs based on multivariate analyses for prediction of inflammatory bowel diseases (IBD) **(A)**; and active clinic stage **(B)** are exhibited.

Then, we included these six ratios and other 4 clinically adopted indicators (CRP, WBC, Hb, Fc) into the ROC analysis. Note that AUC ≥ 0.6 can be seen as a variable could distinguish IBD from healthy controls efficiently, and considered to be taken into further multivariable logistic regression analyses. At a cut-off of 19.10, NPAR differentiated IBD from healthy controls with 57% sensitivity and 88% specificity, of which AUC is 0.74. As Figure 2A showed that NAR, NPAR, AGR, FPR, CRP, and Fc have good diagnostic abilities. Among these variable Fc have the best diagnostic efficiency = 0.823, with 68% sensitivity and 97% specificity, respectively. We evaluated the diagnostic ability of the combination of three or four indicators and found that the AUC of the combined diagnosis was 0.853 and 0.855, which was significantly higher than the AUCs of any indicators separately (*P* < 0.05). The detailed data of the ROC analysis were in the Supplementary materials (Supplementary Table 1). Multivariate analyses were performed to identify the factors that were identified in the univariate analyses. As shown in Figure 3A, NPAR (*P* = 0.016), AGR (*P* < 0.001), and Fc (*P* = 0.023) were identified as significant independent predictors of IBD risk in undiagnosed patients.

### Association between the peripheral blood ratios and IBD disease activity

According to the CDAI, HBI, Mayo Scoring System, and the level of serum CRP, 362 patients with IBD were classified into a group with inactive disease (195, 53.9%), and a group with active disease (167, 46.1%). We assessed the associations between these 20 parameters (6 ratios and 14 clinics frequently used variables) and the IBD stage using logistic regression analysis. Table 3 shows the result of univariate logistic regression analyses, NAR (*P* = 0.047), NPAR (*P* < 0.001), and PNI (<0.001) were found to be significant independent predictors of IBD active risk. Univariate logistic regression factors with a *P* value < 0.05 were eligible for ROC analysis.

**TABLE 3:** Results of univariate logistic regression analysis in patients with active and inactive IBD.

Variable	B	S. E	Wald	P value	OR (95%CI)
NAR*100	0.025	0.013	3.929	**0**.**047**	1.026(1.000–1.052)
NPAR*1,000	0.025	0.005	22.24	**<0**.**001**	1.025(1.015–1.036)
AAPR	-0.003	0.020	0.027	0.870	0.997(0.959–1.036)
AGR	0.045	0.029	2.309	0.129	1.046(0.987–1.107)
FRP*100	0.001	0.001	0.481	0.488	1.000(1.000–1.000)
**PNI**	-0.057	0.014	17.069	**<0**.**001**	0.945(0.920–0.971)
**WBC**	0.104	0.041	6.471	**0**.**011**	1.109(1.024–1.201)
Neu	-0.009	0.040	0.054	0.816	0.991(0.916–1.071)
**Lym**	-0.018	0.005	14.939	**<0**.**001**	0.982(0.974–0.991)
Hb	0.061	0.083	0.534	0.465	1.062(0.903–1.250)
**Hematocrit**	-0.047	0.014	10.409	**0**.**001**	0.954(0.928–0.982)
**RDW**	0.005	0.001	16.846	**<0**.**001**	1.005(1.002–1.007)
Plt	0.003	0.005	0.346	0.557	1.003(0.993–1.013)
**MPV**	0.038	0.006	34.306	**<0**.**001**	1.039(1.026–1.052)
**ESR**	0.023	0.006	16.286	**<0**.**001**	1.023(1.012–1.034)
**Fc**	0.012	0.001	0.770	**0**.**038**	1.188(1.082–1.998)
Bilirubin	0.010	0.009	1.445	0.229	1.010(0.994–1.028)
Creatinine	-0.001	0.006	0.049	0.824	0.999(0.986–1.011)
Uric acid	0.001	0.001	0.220	0.639	1.001(0.998–1.003)
**cCa**	1.795	0.501	12.843	**<0**.**001**	6.021(2.256–16.072)

MPV, mean platelet volume; cCa, calcium-calibration. Bold fonts represented that P value was < 0.05.

The ROC curves for each of the individual test scores are in Figure 2B. As the results demonstrated, NAR (AUC = 0.67), NPAR (AUC = 0.70), and PNI (AUC = 0.70) have a good discrimination ability (AUC ≥ 0.6). Among the other commonly used clinical indicators, ESR(AUC = 0.66), cCa (AUC = 0.62), and Fc (AUC = 0.79) have a good discrimination capacity (AUC ≥ 0.6). The AUC for the combination of NAPR and PNI is 0.70, similar to NPAR or PNI separately. The sensitivity of the combination of NPAR and PNI is 97%, which is higher than that of Fc (72%). The detailed data of the ROC analysis were in the Supplementary materials (Supplementary Table 2).

**FIGURE 4:**
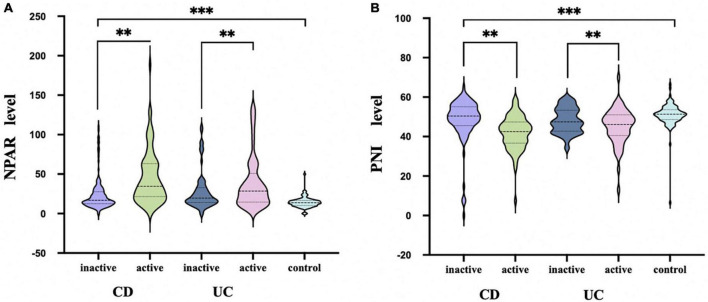
NPAR **(A)** and PNI **(B)** levels in active and inactive disease in comparison with healthy controls. Data are presented as box plots. Statistical analysis: Bootstrap analysis using all active and inactive time points per patient, **P < 0.01, ***P < 0.001.

**FIGURE 5:**
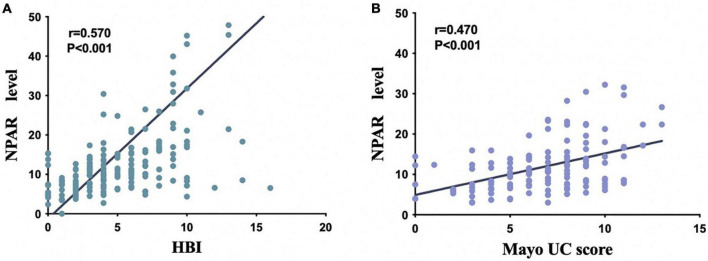
Scatter plot shows the correlation between NPAR and two clinical scores [Harvey Bradshaw index (HBI) in patients with CD **(A)**, Mayo score in patients with UC **(B)**].

Then, multivariate logistic regression analysis was performed to identify independent factors for IBD active stage, including all variables with a P value < 0.05 on univariate logistic regression analysis and AUC ≥ 0.6 on ROC analysis. The *P* values and odds ratios (95% CI) for the individual variables are presented in Figure 3B. As the results indicate, NPAR, PNI, and Fc were independent risk factors for the active stage of IBD. Furthermore, Figure 4A shows a scatter diagram of the relationship between peripheral blood NPAR level and their corresponding clinical stage. The increase in NPAR was associated with disease activity by analysis of the full set of both CD and UC patients. (CD: control vs. inactive CD: *P* < 0.001; control vs. active CD: *P* < 0.001; active vs. inactive CD: *P* < 0.001; UC: control vs. inactive UC: *P* < 0.001; control vs. active UC: *P* < 0.001; active vs. inactive UC: *P* = 0.03). Relationships between PNI variables and CD/UC clinical stage were shown in Figure 4B, the *P* value between groups all < 0.05, considered statistically significant. Finally, Pearson’s linear correlation coefficients were calculated for pairs of NPAR and HBI or Mayo UC scores. The Pearson correlations between change in NPAR and change in HBI or Mayo UC score revealed strong associations, as displayed in Figures 5A, B.

### Predicting the role of NPAR in monitoring IBD-related neoplasia (IBDN)

**FIGURE 6:**
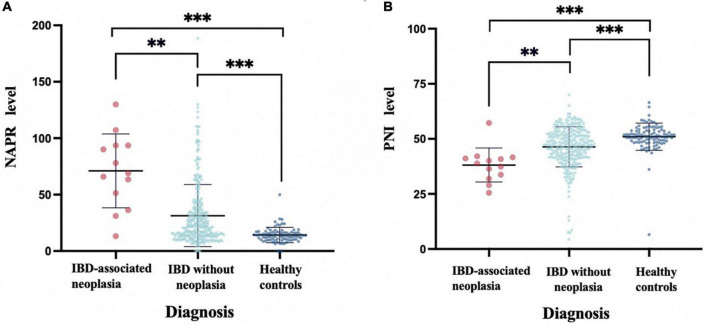
Peripheral blood NPAR levels **(A)** and PNI levels **(B)** in patients with IBD-associated Neoplasia (Dysplasia or CRC) vs. IBD without Neoplasia vs. healthy controls (HC). Boxplots represent median and interquartile ranges for serum NPAR or PNI within each sub cohort, **P < 0.01, ***P < 0.001.

To assess the risk factors for IBDN, 362 patients with IBD were distributed into two groups: IBDN group and IBD without neoplasia group. Neoplasia was detected in at least one colonoscopy and histological results in 10 patients (2.8%); 2(0.6%) of these patients had visible colitis-associated colorectal cancer (CAC). The association between these parameters and the risk of IBD neoplasia is reported in Table 4. As the results demonstrated, there is a significant difference in NPAR (*P* = 0.002) and PNI (*P* = 0.003) between the groups. To facilitate data interpretation, a vertical scatterplot was employed. Figure 6A exhibits the level of NAPR in IBDN groups is significantly higher than IBD without the neoplasia group and Healthy controls (*P* < 0.05). Figure 6B presents the level of PNI is significantly lower than IBD without neoplasia group and Healthy controls (*P* < 0.05). Besides, among clinical parameters, time from diagnosis (*P* = 0.033), the extent of IBD (*P* < 0.001), Intestinal resection in anamnesis (*P* = 0.01), and colonic polyp formation (*P* = 0.012) were significant different between the groups (Table 4).

**TABLE 4:** Analyses of clinical and peripheral blood parameters of Patients with IBD-associated Neoplasia vs. patients without neoplasia.

Variables	IBD-associated Neoplasia (*N* = 12)	IBD without neoplasia (*N* = 350)	*P*- value
Gender			0.370
Male	7(58.3)	234(66.9)	
Female	5(41.7)	116(33.1)	
Age(years)			0.410
≤50	5(41.7)	121(34.6)	
>50	7(58.3)	229(65.4)	
Time from diagnosis(month)			**0**.**033**
≤120	8(66.7)	314(89.7)	
>120	4(33.3)	36(10.3)	
Extent of IBD			**<0**.**001**
Rectal	1(8.3)	23(6.6)	
rectosigmoid	1(8.3)	45(12.9)	
Left colon	2(16.6)	83(23.7)	
Extensive colon	0(0)	139(39.7)	
total colon	8(66.7)	60(17.1)	
Intestinal resection in anamnesis			**0**.**010**
Yes	3(25)	12(3.4)	
No	9(75)	338(96.6)	
infection *Clostridium difficile*			0.171
Yes	4(33.3)	64(18.3)	
No	8(66.7)	286(81.7)	
Abscess or fistula			0.099
Yes	3(25)	32(9.4)	
No	9(75)	318(90.6)	
History of smoking			0.453
Yes	2(16.7)	43(12.3)	
No	10(83.3)	307(87.7)	
HBsAg			0.567
Positive	1(8.3)	23(6.6)	
Negative	11(91.7)	327(93.4)	
Colonic polyp formation			**0**.**012**
Yes	4(33.3)	26(7.4)	
No	8(66.7)	324(92.6)	
NAR*100			0.186
≤16.6	6(50.0)	241(68.9)	
>16.6	6(50.0)	109(31.1)	
NPAR*1,000			**0**.**002**
≤62.1	4(33.3)	269(76.9)	
>62.1	8(66.7)	81(23.1)	
AAPR			0.685
≤0.38	2(16.7)	50(14.3)	
>0.38	10(83.3)	300(85.7)	
AGR			0.551
≤2.05	5(41.7)	118(33.7)	
>2.05	7(58.3)	232(66.3)	
AFR			1.000
≤0.18	4(33.3)	119(34.0)	
>0.18	8(66.7)	231(66.0)	
FPR*100			
≤124.31	6(50.0)	176(50.3)	0.984
>124.31	6(50.0)	174(49.7)	
PNI			
≤42.15	8(66.7)	87(24.9)	**0**.**003**
>42.15	4(33.3)	263(75.1)	

Bold fonts represented that P value was < 0.05.

## Discussion

The rising prevalence, high relapse rates, high morbidity, and the economic burden of treatment, strongly indicate that there is an urgent need to identify convenient and efficient biomarkers to diagnose patients with IBD (21–23). Numerous predicted ratios and scores based on inflammatory associated variables, such as NLR, PLR, CRP/ALB, and modified Glasgow prognostic score (mGPS) have been applied to predict the prognosis of colorectal cancer (24), gastric cancer (25), lung cancer (26) and other cancer. However, a small number of studies have reported the association between inflammation-based ratios and IBD. Thus, we performed this study and found that NPAR and AGR were independently relevant to IBD diagnosis in the whole IBD cohort, while NPAR and PNI were independent risk factors for IBD active stage. NPAR and PNI were also verified to have a difference in the IBDN cohort when compared with IBD without neoplasia group. Moreover, for the first time, our study identifies the best inflammation-based factor among 7 ratios—there is a positive relationship between levels of NPAR and IBD risk, active stage risk, and neoplasia risk.

A literature search manifested that no studies have investigated the role of NAR and NPAR in IBD patients. NAR and NPAR were a convenient marker for systemic inflammation and infection. The difference between these two ratios is the denominator is ALB or pre-albumin. Increased neutrophil percentage and/or decreased pre-albumin levels can attribute to the elevated NPAR levels. ALB is a common indicator of nutritional status, which is synthesized and secreted by the liver. It participates in physiological processes such as the transportation of human nutrients and toxicant metabolism and can resist the invasion of pathogenic microorganisms (27). The serum level of ALB will be decreased under conditions of inflammation and tumor (28). A large community study has reported that low ALB levels often indicate a poor prognosis. Bao et al. (28) have demonstrated that ALB < 40 g/L could predict an unfavorable prognosis in oral cancer patients.

As a diagnostic blood-based marker, the half-life of pre-albumin (1.9 days) is shorter than that of ALB (19 days). Hence, it may provide a more dynamic test and indicate minor changes in a shorter period. Similar to ALB, the serum concentration of pre-albumin is related to nutritional intake, inflammatory state, liver disease, endocrine disease, etc (29). Moreover, the prevalence of malnutrition in IBD patients is high, and increased energy and protein requirements are observed in many patients (30). Numerous studies have explored the association between ALB or pre-albumin and IBD nutrition status. Cho et al. (31) have demonstrated that albumin (*P* < 0.001), and prealbumin (*P* < 0.001) in hair as reliable markers of nutritional status compared to levels in children with IBD. All studies of NAR were focused on the field of coronary artery disease, sepsis, or septic shock in recent years (32, 33). In this study, we first reported that circulating NPAR harbored the best diagnosis and prediction ability compared with the other inflammatory ratios. And the combination of NAR, NPAR, and AGR presented a higher predictive value than Fc, with AUC of 0.853 and 0.823, respectively.

Beyond diagnostics, our research also has explored the utility of non-invasive markers, based on inflammatory ratios, in predicting disease clinic activity. It is well-known that ESR and fecal calprotectin (FC) can be used to evaluate disease status in patients with IBD (34). In our study, we validate the diagnosis ability of Fc and ESR, with sensitivities of 72.0 and 42.0% and specificities of 84.0 and 88.0%, respectively, consistent with previous studies (35). Moreover, NPAR and PNI ratio were statistically significant parameters capable of discriminating the disease activity from inactive after multivariable logistic analysis. Takaki et al. (36) have suggested that ALB correlated negatively with disease activity in UC. Another research shows that ALB was significantly lower in patients with active disease (*P* < 0.01), and C-reactive protein/albumin ratio (CAR) is a useful biomarker for identifying disease activity in patients with IBD (AUC = 0.829) (37), which is similar to our study results.

Recently, it has been reported that patients with IBD have a 1.7-fold increased risk of CRC (38). More recent studies have focused on identify prognostic factors for advanced colorectal neoplasia (aCRN, high-grade dysplasia (HGD), or CRC) in patients with IBD. At present, the mechanism for this is still unclear, but, certainly, chromosome instability (CIN), microsatellite instability (MSI), inflammatory factors, oxidative stress, and DNA methylation are involved in this process (39, 40). Frank et al. (41) have reported that neoplasia was detected in only four patients (7.5%) during 10-year surveillance. The low yield and lack of clinical consequences raise questions about the necessity of routine endoscopy use during IBD surveillance. There is an unmet need to identify the high-risk CRC cohort. As widely studied in recent years, clinical neoplasia risk factors like extensive disease, colon segment resection, and inflammatory polys (42) have been proven to have a significant difference in patients with or without neoplasia in our study. Voluminous and diverse literature reported that IBD could trigger choric inflammation and increase the risk of CRC (39). Ying et al. (43) have reported that FPR is superior to the other inflammatory biomarkers as a useful recurrence indicator in CRC patients with excellent prognostic ability. To our date, we found that level of NPAR and PNI has a significant difference between the two groups, although there are still no regression results to validate that these indicators are ideal biomarkers for IBD neoplasia. Peripheral blood ratios based on inflammatory parameters are just starting to blossom but manifest great promise for future clinic practice, which may potentially increase the yield of surveillance by adding these biomarkers to the routine assessment of endoscopy. Thus, large-scale, multicenter clinical studies are needed to identify the risk factor of IBDN and construct a predictive model for IBDN.

One of the highlights of our study was to systematically explore the function of the ratios based on inflammatory indicators to assess IBD risk, disease activity, and potential neoplasia. All these ratios are convenient, and easy to obtain without additional examination, which may help clinicians obtain information to make personalized treatment decisions. Another one of the highlights of our study was to conduct both univariate, multivariable regression analysis and ROC analysis to eliminate possible confounding bias risks that we can find the actual significance biomarker in the diagnosis of IBD and predictive IBD activity.

However, limitations should be addressed as follows. First, the number of included patients is relatively small, it mat limits the power to dissect factors. Second, some potentially adorable markers for IBD diagnoses, such as auto-antibodies (Anti-neutrophil cytoplasmic antibodies, ANCA; Anti-glycoprotein 2, Anti-GP2), were not evaluated in our study, as only some included patients underwent that test. Hence, we cannot make a comparison to these indicators, which may enhance the reliability of our results. Third, the gap between the number of IBDN group and the IBD without neoplasia group is great, which might have biased the results. In the future, studies with larger IBDN sample numbers should be taken to further clarify the independent risk factor of IBDN.

## Conclusion

We demonstrated NPAR, and AGR presented a good diagnosis value in differentiating between IBD and healthy controls. Furthermore, NPAR and PNI show promise as inflammatory-based biomarkers in predicting the disease phase in IBD, but also potentially associate with inflammation to carcinoma in patients with IBD. Taken together, our findings strongly suggested that the NPAR could serve as a simple, non-invasive, and excellent discriminator for clinical diagnosis, predictive activity, and prognosis in IBD. Moreover, combining different indicators may be valuable in improving the performance of disease evaluation.

## Data availability statement

The raw data supporting the conclusions of this article will be made available by the authors, without undue reservation.

## Ethics statement

The study was conducted according to the guidelines of the Declaration of Helsinki, and approved by the Ethics Committee of Renmin Hospital of Wuhan University (No. 2018K-C089). The patients/participants provided their written informed consent to participate in this study.

## Author contributions

JL and YG: study conception and design and manuscript review and editing. JP and JL: data collection. JL: statistical analysis. JP: manuscript draft. All authors contributed to the article and approved the submitted version.
