# Supplementary material for: The value of 7 peripheral blood serum ratios in diagnosis and prediction of disease activity of patients within inflammatory bowel disease individuals
Source: Front Med (Lausanne). 2023 Apr 5;10:1122005. doi: 10.3389/fmed.2023.1122005 (PMC10113552; doi:10.3389/fmed.2023.1122005)
Supplement: Supplementary file 1 [file Table_1.DOCX]

**Supplement Table 1** Accuracy of NPAR and other markers in differentiating IBD from healthy controls.

| Variables | AUC (95% CI) | S. E | Cut-off | Sensitivity | specificity |
| --- | --- | --- | --- | --- | --- |
| **NAR*100** | **0.694(0.604-0.784)** | 0.046 | 12.12 | 0.40 | 0.92 |
| **NPAR*1000** | **0.744(0.683-0.785)** | 0.026 | 19.10 | 0.57 | 0.88 |
| AAPR | 0.379(0.276-0.482) | 0.052 | 0.75 | 0.10 | 0.78 |
| **AGR** | **0.699(0.639-0.721)** | 0.037 | 2.41 | 0.57 | 0.99 |
| **FRP*100** | **0.684(0.628-0.740)** | 0.028 | 107.30 | 0.56 | 0.77 |
| PNI | 0.408(0.312-0.503) | 0.049 | 55.94 | 0.13 | 0.90 |
| **CRP** | **0.765(0.706-0.825)** | 0.031 | 6.90 | 0.43 | 0.95 |
| WBC | 0.590(0.530-0.651) | 0.047 | 7.60 | 0.31 | 0.91 |
| Hb | 0.265(0.259-0.448) | 0.029 | 149.5 | 0.10 | 0.73 |
| **Fc** | **0.823(0.768-0.879)** | 0.028 | 47.2 | 0.68 | 0.97 |
| **NAR+NPAR+AGR** | **0.853(0.816-0.891)** | 0.019 | 0.80 | 0.97 | 0.72 |
| **NAR+NPAR+AGR+FRP** | **0.855(0.818-0.892)** | 0.019 | 0.97 | 0.75 | 0.93 |

**Supplement table 2** Accuracy of NPAR and other markers in differentiating active from inactive IBD.

| Variables | AUC (95% CI) | S. E | Cut-off | Sensitivity | specificity |
| --- | --- | --- | --- | --- | --- |
| **NAR*100** | **0.670(0.608-0.732)** | 0.032 | 10.05 | 0.67 | 0.64 |
| **NPAR*1000** | **0.697(0.683-0.785)** | 0.032 | 21.30 | 0.68 | 0.63 |
| **PNI** | **0.701(0.632-0.749)** | 0.030 | 46.23 | 0.70 | 0.63 |
| **WBC** | **0.609(0.544-0.673)** | 0.034 | 5.68 | 0.72 | 0.50 |
| Lym | 0.401(0.336-0.446) | 0.033 | 1.64 | 0.29 | 0.58 |
| Hematocrit | 0.381(0.317-0.446) | 0.033 | 83.2 | 0.56 | 0.28 |
| RDW | 0.447(0.380-0.514) | 0.034 | 45.15 | 0.28 | 0.71 |
| MPV | 0.343(0.280-0.406) | 0.032 | 10.35 | 0.35 | 0.46 |
| **ESR** | **0.656(0.588-0.723)** | 0.036 | 41.5 | 0.42 | 0.86 |
| **cCa** | **0.617(0.552-0.683)** | 0.033 | 2.27 | 0.45 | 0.77 |
| **Fc** | **0.794(0.745-0.842)** | 0.024 | 82.3 | 0.72 | 0.88 |
| **NPAR+PNI** | **0.702(0.652-0.738)** | 0.021 | 0.80 | 0.97 | 0.72 |
